# The Applicability of the Poincaré Plot in the Analysis of Variability of Reaction Time during Serial Testing

**DOI:** 10.3390/ijerph18073706

**Published:** 2021-04-02

**Authors:** Elena Ioana Iconaru, Manuela Mihaela Ciucurel, Luminita Georgescu, Mariana Tudor, Constantin Ciucurel

**Affiliations:** 1 Department of Medical Assistance and Physical Therapy, University of Pitesti, 110040 Pitesti, Romania; mariana.tudor@upit.ro (M.T.); constantin.ciucurel@upit.ro (C.C.); 2 Department of Psychology and Communication Sciences, University of Pitesti, 110040 Pitesti, Romania; manuela.ciucurel@upit.ro; 3 Department of Physical Education and Sport, University of Pitesti, 110040 Pitesti, Romania; luminita.georgescu@upit.ro

**Keywords:** reaction time, variability, nonlinear dynamics, health status, anxiety, aging

## Abstract

(1) Background: This study aims to put into evince the relationship between the variability of the reaction time (RT) during repeated testing, expressed through indicators extracted by the Poincaré plot method, and the age of the participants, their self-reported health (SRH), and level of perceived anxiety. (2) Methods: The study was performed using computerized RT testing software. An observational cross-sectional study was performed on a group of 120 subjects (mean age 42.33 ± 21.12 years), sex ratio men to women 1.14:1. Data were processed through descriptive and inferential statistics. The Poincaré plot method was applied in the analysis of the RT series of data, by calculating the indicators SD_1_, SD_2_, SD_1_/SD_2_, and area of the fitting ellipse (AFE) (3) Results: We provided evidence of the excellent reliability of the web-based RT serial testing (Cronbach’s Alpha 0.991) with this sample group. Our results showed that age is an important predictor for mean values of RT, while SD_1_, SD_2_, and AFE indicators are for SRH (*p* < 0.01). (4) Conclusions: the variability of RT, expressed by the Poincaré plot indicators, reflects the health status rather than the aging of the subjects and is barely influenced by their level of anxiety.

## 1. Introduction

Reaction time (RT), a measure of the time response to a specific stimulus, is a test with multiple clinical applications, with regard to function and health [1]. Different types of RT have been proposed for visual, auditory, or tactile stimulation. Thus, its practical utility has been demonstrated especially in geriatrics and gerontology [2], psychology and related fields [3], neurology [4], sports medicine [5], etc., Ref. [6]. 

In addition to the classic computerized testing of RT, with a small number of repetitions, more recently cognitive research has stood out on the indicator variability during serial testing to theoretically and clinically relevant manipulations [7]. The variability of specific biomarkers during the aging process, with explanations derived from the study of the entropy of human development, is another exciting topic of discussion. This type of research is based on the concept of increasing entropy with age, which can be expressed in terms of changing the repeatability, uniformity, predictability, and homogeneity of certain biomarkers during repeated testing in young adults compare to older adults [8]. Thus, for cognitive tasks, an increase of variability correlated with aging and cognitive functioning was also highlighted [9]. However, it should not be neglected that RT is negatively influenced by various types of pathologies associated with the aging process, especially of brain disease type [10]. Also, the relationship between RT and anxiety level is the subject of much debate [11], but the effects of anxiety on RT variability are unclear [12].

The study of the variability of some physiometric indicators, from a series of data extracted during repeated tests, can be accurately performed using Poincaré plot analysis. The Poincaré plot is a geometrical technique that draws a return map of a time series of data and quantifies the recurrence, self-similarity, or periodicity of state variables of systems [13]. The resulting plot is an ellipse, which can provide the following important descriptors: SD_1_ (the minor axis of the ellipse, indicating the short-term variability of the time series of data), SD_2_ (the major axis of the ellipse, indicating the long-term variability of the time series of data), the ratio SD_1_/SD_2_ (which measures the relative balance between short- and long-term variabilities of the time series of data), and the area of the fitting ellipse (AFE) [14,15,16,17]. Through the Poincaré plot method, we can transform an initial qualitative analysis of a nonlinear distribution of data into a quantitative analysis in terms of linear statistics, by calculating the specific indicators [8].

The application of the Poincaré plot in studying the dynamics of fluctuations in physiological rhythms has received much attention in recent years in the biomedical field because it offers a geometrical representation of nonlinear dynamic models [17,18]. This method has large applications for the study of the heart rate variability [19], but also of other types of parameters: respiratory rate, blood pressure, blood glucose, electroencephalographic and polysomnography paths, plethysmography, electromyography or electrohysterography data, biomechanical indicators (gait, body center of pressure or hand grip strength variability) [8]. Recently, the method has been proposed for the study of the chronotropic regulation of the heart because it allows identifying patterns in non-stationary variables as heart rate, which involves the dynamic interaction between the multiple physiologic mechanisms [20].

However, the need to record longer strings of data, usually of the type of biosignals or biomarkers, may raise certain methodological difficulties. Regarding a possible application of the Poincaré plot method in the case of RT determination, we could not identify such research in our literature review. Classically, RT determination involves various number of trials (5–40) to draw reliable results [21]. The way of analyzing the RT variability from the perspective of the concept of nonlinear dynamics has been less the object of researchers’ attention. To our knowledge, this is the first study to quantify the self-similarity features of nonlinear dynamics of RT during repetitive tasks. To tackle this problem, we designed a novel design of computerized testing of RT, with repetitive tasks to obtain a sufficient number of data, suitable for analysis using the Poincaré plot method. Thus, we wanted to establish the relationship between the Poincaré plot indicators, the age of the participants, their state of health, and level of perceived anxiety. In this study, we hypothesized that RT variability is related to the age, health status, and anxiety level of the subjects, and we used the Poincaré plot method to test this hypothesis. The emerging pattern of the RT time series would provide valuable insight into the temporal dynamics of a complex cognitive task, in relation to the mentioned parameters.

## 2. Materials and Methods

### 2.1. Participants and Type of Study

Ethics approval for the research was obtained from the Ethics Committee of the Research Center for Promoting Excellence in Professional Training, University of Pitesti (reference number 1366/11 June 2020). An observational cross-sectional study was performed on a group of 120 subjects (mean age 42.33 ± 21.12 years), sex ratio men to women 1.14:1. The subjects were selected with the help of field operators, students at the University of Pitesti. The operators were previously trained on the computerized RT testing procedure. They initially participated directly as subjects in the research, and then each of them performed the supervised testing of 4–6 other subjects (family members or friends).

All participants, at the time of testing, had a normal or corrected-to-normal vision (by self-report) and did not have a significant pathological history (chronic or acute neuromotor pathology, recent injuries, cognitive disorders, or other medical conditions that could interfere with RT testing). For each individual, we required online informed consent to participate in the study under the ethics of research on human subjects. 

### 2.2. Data Acquisition

The study was performed using a computerized RT testing software, which was built on a web platform. Thus, for online data collection, the software PsyToolkit (https://www.psytoolkit.org/) (accessed on 1 October 2020) was used [22,23]. The software program for PC/laptop consists of two distinct parts: a short introductory questionnaire and the experiment.

The questionnaire has a first section with informed consent, then 5 closed questions with coded answers related to age (years), sex (1 = male, 2 = female), professional status (1 = pupil/student, 2 = employee, 3 = unemployed, 4 = retired, 5 = household), self-reported health (SRH—on a 5-point scale ranging from 1 = excellent, 2 = very good, 3 = good, 4 = satisfactory, 5 = poor), self-reported anxiety (SRA—on a 5-point scale ranging from 1 = not at all anxious, 2 = slightly anxious, 3 = moderately anxious, 4 = very anxious, 5 = extremely anxious), and respectively the dominant hand (with which it writes—1 = right or 2 = left).

The SRH is considered to be a valid indicator for the prediction of health outcomes [24]. The chosen variant of quoting the answers is most widely used in the US [25]. For the self-perceived level of anxiety, we used a short scale, with 5 levels, frequently used in the field [26].

**Figure 1:**
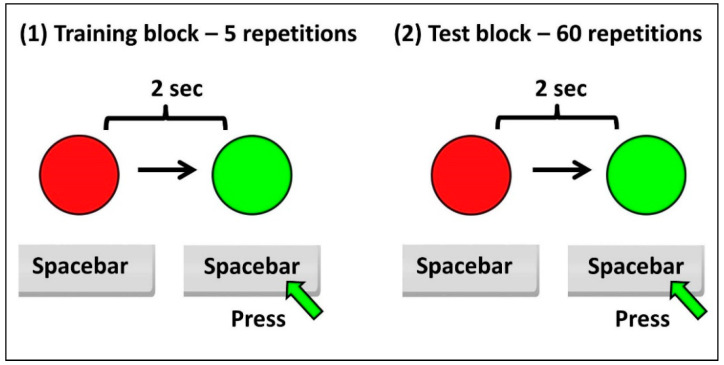
Schematic of the RT experiment. (1) Training block- 5 repetitions; (2) Test block-60 repetitions.

The experiment (Figure 1) comprises two blocks of RT testing for visual stimuli: one for information and training (with 5 repetitions of RT), and then the test itself (with 60 repetitions of RT). The total duration of the experiment is on average 3–5 min. RT testing is performed by pressing the Spacebar key as quickly as possible with the dominant hand after changing the color of a circle on the monitor screen from red to green. We chose this graphic variant because it is derived from a usual model, the traffic light. The interval between changing colors was set to 2 s, and the maximum allowed response time was 3 s. If the subject does not press the Spacebar key within 3 s after the color of the circle has been changed in green, the answer is no longer taken into account. The selection of subjects, who correctly answered and completed the experiment, was based on compliance with this principle. 

At the end of the second block, the average value of the RT for the 60 successive tests is displayed on the screen. The recommended conditions for performing the test referred to a quiet room to minimize distractions, attention on the task, without environmental disturbances. Regarding the hardware delay (linked to the screen’s vertical refresh rate and the Spacebar click) and the web-platform timing performance, which can inflate true RT values, most authors consider the response-time lags as being negligible, in most configurations [27].

### 2.3. Outcomes and Statistical Analysis

Data were imported and processed using IBM SPSS 20.0 software (IBM Corp., Armonk, NY, USA) [28] through descriptive statistics (mean, standard deviation, Shapiro–Wilk test for testing the normality of data, Cronbach’s alpha test for the internal consistency of the RT test, Pearson’s correlations and Spearman’s rank correlation between variables) [29] and inferential statistics (t-test for unpaired groups, simple linear, ordinal and multiple regression, after checking the required assumptions). The results are presented as mean ± SD.

We also applied the Poincaré plot method in the analysis of the RT series of data, by calculating the indicators SD_1_, SD_2_, SD_1_/SD_2_, and AFE. The formulas for the mentioned parameters are the following [14,15,16,30]:

$${\mathrm{SD}}_{1}=\frac{\sqrt{2}}{2}*\mathrm{SD}\left(x_{n}-x_{n+1}\right)\;{\mathrm{SD}}_{2}=\sqrt{2\mathrm{SD}{\left(x_{n}\right)}^{2}-\frac{1}{2}\mathrm{SD}{\left(x_{n}-x_{n+1}\right)}^{2}}$$

where SD(*x_n_* − *x_n_*_+1_) represents the standard deviation of the time series of the successive differences *x_n_* − *x_n_*_+1_ and SD(*x_n_*) the standard deviation of the time series *x_n_*.

$$\mathrm{AFE}=\pi *{\mathrm{SD}}_{1}*{\mathrm{SD}}_{2}$$

## 3. Results

### 3.1. Respondents

A total number of 120 participants were finally selected for statistical data processing (a recruitment rate of our web-based study of 78.43%). Eligibility was based on completing the experiment, taking into account the inclusion and exclusion criteria of the subjects in the study. For the final group of participants, no missing data and no significant outliers were observed. The fact that most of the subjects have completed the experiment justifies its accessibility in the online form. It is also worth noting the easiness of gathering and building the database.

### 3.2. Summary Characteristics of the Study Participants

The results are synthetically presented as descriptive statistical indicators in Table 1.

It is observed that the mean age of the group of subjects was 42.33 ± 21.12 years (range 18–88 years), 53.33% men and 46.67% women. Overall, the SRH mean level (mean score 2.37 ± 0.90) indicated a very good—good health status of the group, with no anxiety—mild anxiety (mean score 1.71 ± 0.89 for SRA). More exactly, 19.2% of the subjects reported excellent health, 34.2% very good health, 37.5% good health, and 9.2% satisfactory health. Regarding anxiety, 52.5% of the subjects reported no anxiety at all, 30% mild anxiety, 11.67% moderate anxiety, and 5.83% severe anxiety. For the mean RT variables and indices derived from the Poincaré plot, we applied the Shapiro-Wilk test to determine the type of data distribution and we obtained a normal distribution only for the SD_1_ indicator. We also calculated the statistical indicators for the subgroups of men and women (Table 2), because many classical studies took into account the sex factor in the interpretation of RT values.

Next, we determined the statistically significant differences between the subgroups of men and women in relation to these variables, by repeatedly applying the t-test for independent samples (Table 3).

It was found that the differences between the means of the analyzed variables in the subgroups of men and women were not statistically significant.

### 3.3. Reliability of the RT Serial Test

To assess the internal consistency of the RT serial test we applied the Cronbach’s Alpha. As a result, the Cronbach’s Alpha was 0.991 which is considered to reflect excellent reliability.

### 3.4. Correlation Analysis for the Recorded Variables

The next step was to determine Pearson’s correlation coefficient between the continuous variables, respectively Spearman’s rank correlation coefficient in the case of the ordinal variables, to later determine the inferential character of the main obtained correlations [29]. Thus, from Table 4 of interest are the high positive correlation between RT and age (0.79), as well as the moderate positive correlations between SRH and the indicators SD_1_ (R = 0.43), SD_2_ (R = 0.41), and AFE (R = 0.44). It should be noted that the high correlation coefficients recorded between the indicators specific to the Poincaré analysis, are explainable by the fact that they are obtained from intricate formulas.

### 3.5. Simple Linear Logistic Regression Analysis

Starting from the correlations obtained between the investigated continuous variables, we repeatedly performed a standard linear regression analysis between the age and the Poincaré parameters. Even if the variables are non-normal distributed, linear regression can successfully be applied in studies of large sample sizes [31]. The results of simple linear regression analysis which was conducted to determine the effect of age (the predictor variable) on the parameters measured (the outcome variables) are presented in Table 5.

**Figure 2:**
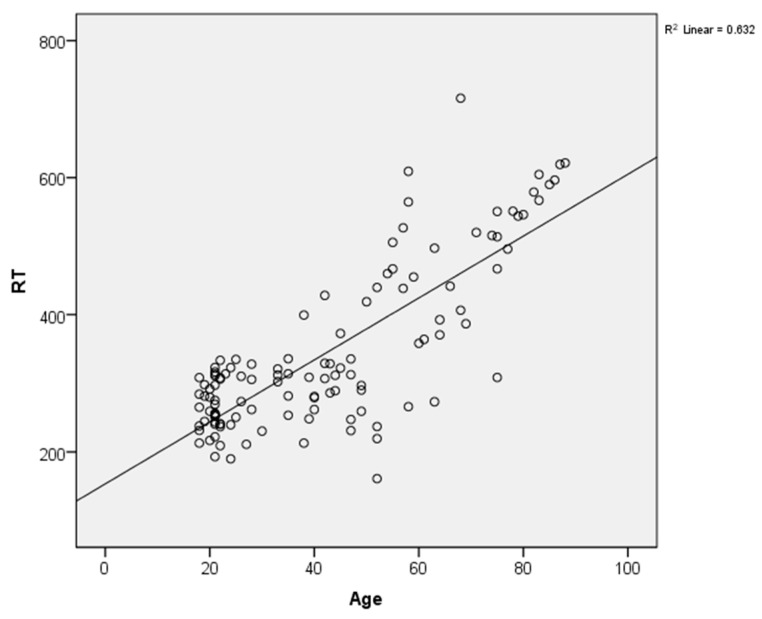
Simple linear regression analysis representing the association of age with reaction time (RT).

We found that three of our regression models are statistically significant (associations between age and RT, age and SD_1_, age and SD_1_/SD_2_) and therefore a certain part of the variability of the dependent variables is explained by the independent variable (age). The best regression model that significantly predicts the dependent variable was in the case of the association between age and RT (Figure 2). Adjusted R square is also an estimate of the effect size, our results indicating a medium effect size for the effect of age on RT, according to Cohen’s (1988) classification [32].

### 3.6. Ordinal Regression Analysis

Since SRH is an ordinal variable, we performed an ordinal regression analysis, considering as continuous independent variables the parameters extracted from Poincaré plot, and as dependent variable SRH. Thus, we wanted to determine which of our independent variables (if any), as predictors, have a statistically significant effect on the dependent variable (SRH).

From the repeated univariate ordinal logistic regression (Table 6), the statistically significant chi-square statistic indicated that each final model gives a significant improvement over the baseline intercept-only model, except for the SD_1_/SD_2_ variable. The pseudo R-square Nagelkerke values indicate that some predictors explain a relative proportion of the variation between subjects in their SRH. The best model of prediction is based on AFE, this parameter explaining 25% from variance in SRH, followed, in order, by the models based on SD_1_ (21% of SRH variance) and SD_2_ (20% of SRH variance). From the analysis of parameter estimates, we also determined the same independent variables with a statistically significant effect on the dependent variable (SRH). In conclusion, there is a link between each considered variable (except SD_1_/SD_2_) and SRH, each of these variables explaining a certain part of the variance in SRH.

### 3.7. Multiple Regression Analysis

Based on the previously obtained results, we considered it useful to perform a multiple regression analysis (Table 7) to understand whether some Poincaré parameters (dependent variables RT, SD_1_, SD_2_, and respectively AFE) can be predicted based on age and SRH (independent variables).

It is observed that the multiple regression does not significantly improve the considered predictive models, the values R, R square, and adjusted R square being comparable to those obtained from the simple linear regression analysis. In other words, age significantly influenced RT, and the indicators SD_1_, SD_2_, and AFE influenced SRH, but the combined effect of age and SRH on each dependent variables did not bring significant additional influences.

## 4. Discussion

Results of our study provided evidence of the excellent reliability of the web-based RT serial testing (Cronbach’s Alpha 0.991) with this sample group, as a measure of the similarity of the RT response across trials. On the other hand, applying Poincaré plot quantification for assessing RT variability in the experimental design, we found that age is an important predictor for mean values of RT, and indicators SD_1_, SD_2_, and AFE for SRH (*p* < 0.01). In other words, the long-term and short-term variability of the data, but also the ellipse fitting process, indicated a tendency of increasing uncertainty in the conditions of the deterioration of the health condition of the subjects. Instead, the influence of age was notifiable on the mean RT and less on the parameters that reflect the variability.

Our results regarding the correlation between mean RT and age are consistent with the previous reports, which highlighted the fact that older participants have greater diversity in RT performance than younger adults, and simple RT becomes slower and more variable with age [9,33]. However, in our study, the RT variability, expressed by indicators, extracted from Poincaré plot analysis, did not correlate with the age of the subjects. This result is consistent with a study on RT and choice RT performance during aging, which showed that older adults are more variable than younger adults in choice RT performance, but not simple RT performance [34]. In our case, we also considered the dynamics of simple RT during repetitive testing in relation to age and we put into evince a statistically significant high linear correlation between the two parameters, with a medium effect size (*p* < 0.001). On the other hand, some authors, in the conditions of different experimental designs (with a small number of repetitions of the RT test and with random intervals between stimuli), highlighted an increased RT mean and variation with increasing, but a non-linear relationship between RT and age [35].

Another interesting study that can be comparatively analyzed refers to the mean increase of simple RT with age, but in the case of auditory stimuli, with a rate of approximately 0.5 ms/year, consistent with the hypotheses of slowing of behavior and increasing within-participant variability, as a continuous age-associated process [36]. From the point of view of the incriminated mechanisms, even important aspects of the relationship remain unclear, the age-related slowing in visual RT latencies is explained through delays in response selection, motor execution [27], sensory visual processing [37], and parallels age-related declines in areas of cognitive functioning [38]. Also, the slowing of RT during aging may be exaggerated by increasing difficulty task or complexity, and disjunctive RT at high tones auditory stimulus increase more linear with age than simple RT [36].

In conclusion, for our analysis of the relationship between mean RT and age, we found that by repeating the task 60 times we highlighted a high positive linear correlation between the two parameters for subjects between 10 and 88 years old, although traditionally, studies indicate the nonlinearity of the association of the mentioned variables, with significant changes after 50 years old [38]. We have obtained a significant linear relationship between age and mean RT values by increasing the complexity of the testing design, which requires a subject’s focus on a longer task, with greater involvement of higher cortical functions.

In contrast, the obtained correlations between Poincaré indicators, which reflect RT variability during testing, and age are minor, although many authors have reported different results [9]. Thus, it was proved that the older subjects require more training to become familiar with the RT task and short tests may disproportionately increase variance in older subjects [27]. According to this interpretation, in the case of our repeated testing design, the variability of RT can be blurred by repeating the task 60 times.

Regarding the analysis of the results according to sex, we could not highlight significant differences between the subgroups of men and women in relation to the considered variables. It should be mentioned that the data in the literature are contradictory related to this subject depending on the experimental design, and there are no clear predictive models of RT according to age and sex [36]. However, most authors have revealed that men are often found to have faster and less variable simple RT across the life span than do women, but there are no sex differences in mean complex RT, with multiple choice [36,39,40]. Also, under the conditions of a particular task of visual choice RT, the cognitive performance of women is superior, women having a faster decision time than men, while men have a faster movement time [41]. In our case, we can appreciate that the repetitive test involved a complex task of cognitive processing, and the interpretation of the results becomes consistent with the mentioned bibliographic sources.

Interesting are also the results concerning the SD_1_, SD_2_, and AFE indicators, which had a moderate correlation with SRH, while mean RT was low correlated with the same parameter. To interpret these results, we started from the concept of entropy, which reflects the thermodynamic level of organization and functioning of the human body, as an open biological system, in physiological or pathological allostatic states [42,43]. Thus, in the case of the human body, increasing entropy leads to the constant loss of usable energy, as a theoretical formulation for progressive disorder and randomness in the cells [44]. From this perspective, the extracted indices from the Poincaré plot analysis of the non-linear dynamics of RT expressed a greater variability of the complex control mechanisms involved, in the conditions of altered health. Thus, they can be considered as cognitive biomarkers of health, and at least part of their variation can be explained by the concept of nonlinear dynamics.

Other authors have shown that intraindividual variability of RT represents a cognitive marker of neurobiological disturbance, and has been associated with dementia and mortality in old age [45]. Furthermore, this variability seems to be influenced by the healthy lifestyle, in terms of a relationship with biological markers, such as forced expiratory volume at one second, grip strength, and vision [34]. In contrast, many studies have undoubtedly shown that RT values are negatively influenced by various pathological conditions [46,47] and positively by the active lifestyle and training for physical fitness [39,48]. Consistent with the above-mentioned information, the novelty of our study refers to the relatively simple method of determining the RT variability by the Poincaré plot method, bringing to light the concept of nonlinear dynamics, which can explain, at least partially, our results.

A final analysis of the results considered the relationship between anxiety and RT. Thus, we showed very low correlations between the SRA level and the extracted parameters from the Poincaré plot analysis (Spearman correlation coefficients below 0.25). Classically, it is considered that anxiety slowed RT due to impaired cognitive performance [11]. Meantime, there are controversial opinions that claim that anxiety may benefit performance in low cognitively demanding tasks [49,50]. Instead, much clearer is the inverse relationship according to which a slower processing speed represents a risk factor for the development of psychological distress [51]. In our case, the low recorded correlations can be explained by the fact that a repetitive test, which is not very demanding, tends to attenuate the influence of anxiety. We can also consider that the repetition of the task leads to the stimulation of the learning processes of the task. Nevertheless, the fact that the tests took place at the participants’ homes, in a familiar environment, with usual devices and an accessible way of working, can reduce the influence of anxiety on the recorded results. This makes it appropriate to use the proposed testing method in people with emotional disorders, for whom anxiety can negatively affect the accuracy of the results.

The present study provided evidence for the validation of the proposed experimental design, which has the potential to be applied as a feasible alternative to traditional testing methods. Practically, it offers the possibility of easy determination of variability of RT, as a cognitive biomarker of subjects’ health status and age. Our results support the idea that examining the variability of RT during serial testing through the Poincaré plot method brings more information about the subjects’ neurobiological status, by reference to their health and age. As a result of this study, new directions of research may emerge, such as the validation of nomograms for RT variability depending on age, the determination of the impact of certain pathologies on RT variability, the identification of risk groups for pathological aging, the study of longevity from the entropic perspective of cognitive processes.

Mainly, the methodological limitations of the study refer to the way of data collecting, which raises the issue of sincerity and subjectivism of the participants in choosing the appropriate answers to the preliminary questionnaire, but also their involvement in performing the required tasks. Also, in the study of RT variability during testing we did not consider determining the effects of boredom, task learning, and performance increasing through repeated training. Moreover, certain influences that can be discussed are related to the abilities of the subjects to use the PC, the performance of the PC, and the quality of the Internet connection.

## 5. Conclusions

The results lead to a conclusion that the age of the subjects correlated more with the mean RT, obtained by serial testing, and much less with the RT variability. In contrast, the health status of the subjects correlated better with RT variability than with mean RT. The relationship between RT variability and health status becomes evident, in terms of the extracted Poincaré indicators. Thus, the RT variability, expressed by the Poincaré plot indicators, reflects the health status rather than the aging of the subjects and is barely influenced by their level of anxiety.

## Figures and Tables

**Table 1:** Statistic indicators for investigated parameters in the experimental group (*n* = 120).

Variable	Age Years	SRH	SRA	RT ms	SD_1_ ms	SD_2_ ms	AFE ms^2^	SD_1_/SD_2_
Mean	42.33	2.37	1.71	344.59	81.11	85.68	24604.19	0.96
SD	21.12	0.90	0.89	119.94	31.68	34.47	17178.73	0.21

Note: *n*: number of subjects; SRH = self-reported health; SRA = self-reported anxiety; RT = reaction time; SD: standard deviation; AFE = area of the fitting ellipse.

**Table 2:** Statistic indicators for investigated parameters in the subgroups of men (*n* = 64) and women (*n* = 56).

Variable		Age Years	SRH	SRA	RT ms	SD_1_ ms	SD_2_ ms	AFE ms^2^	SD_1_/SD_2_
**men**	**Mean**	41.83	2.36	1.73	333.85	80.95	85.31	24443.93	0.96
	**SD**	21.64	0.86	0.95	127.59	32.12	33.92	16814.49	0.21
**women**	**Mean**	42.89	2.38	1.68	356.86	81.31	86.10	24787.34	0.97
	**SD**	20.68	0.95	0.83	110.41	31.46	35.39	17736.77	0.22

Note: *n*: number of subjects; SRH = self-reported health; SRA = self-reported anxiety; RT = reaction time; SD: standard deviation; AFE = area of the fitting ellipse.

**Table 3:** The *t*-test values, the thresholds of statistical significance *p* for differences between men and women subgroups’ means.

Parameter	*t*	*p*
Mean age men (*n* = 64) versus mean age women (*n* = 56)	0.27	0.78
Mean SRH men (*n* = 64) versus mean SRH women (*n* = 56)	0.09	0.92
Mean SRA men (*n* = 64) versus mean SRA women (*n* = 56)	0.34	0.73
Mean RT men (*n* = 64) versus mean RT women (*n* = 56)	1.05	0.29
Mean SD_1_ men (*n* = 64) versus mean SD_1_ women (*n* = 56)	0.06	0.95
Mean SD_2_ men (*n* = 64) versus mean SD_2_ women (*n* = 56)	0.12	0.90
Mean AFE men (*n* = 64) versus mean AFE women (*n* = 56)	0.11	0.91
Mean SD_1_/SD_2_ men (*n* = 64) versus mean SD_1_/SD_2_ women (*n* = 56)	0.15	0.88

Note: SRH = self-reported health; SRA = self-reported anxiety; RT = reaction time; SD: standard deviation; AFE = area of the fitting ellipse; *n*: number of subjects.

**Table 4:** Matrix of correlations between the recorded variables (*n* = 120).

Variable	Age	Sex	Profession	SRH	SRA	RT	SD_1_	SD_2_	AFE	SD_1_/SD_2_
**Age**	1.00 ^a^									
**Sex**	0.05 ^a^	1.00 ^a^								
**Profession**	0.74 ^a^	−0.06 ^a^	1.00 ^a^							
**SRH**	0.30 ^a^	0.02 ^a^	0.36 ^a^	1.00 ^a^						
**SRA**	0.21 ^a^	−0.01 ^a^	0.20 ^a^	0.41 ^a^	1.00 ^a^					
**RT**	0.79 ^b^	0.18 ^a^	0.57 ^a^	0.24 ^a^	0.20 ^a^	1.00 ^b^				
**SD_1_**	0.23 ^b^	−0.01 ^a^	0.41 ^a^	0.43 ^a^	0.25 ^a^	0.42 ^b^	1.00 ^b^			
**SD_2_**	0.09 ^b^	0.01 ^a^	0.25 ^a^	0.41 ^a^	0.15 ^a^	0.26 ^b^	0.81 ^b^	1.00 ^b^		
**AFE**	0.13 ^b^	0.01 ^a^	0.35 ^a^	0.44 ^a^	0.22 ^a^	0.32 ^b^	0.91 ^b^	0.95 ^b^	1.00 ^b^	
**SD_1_/SD_2_**	0.32 ^b^	0.01 ^a^	0.18 ^a^	−0.01 ^a^	0.19 ^a^	0.36 ^b^	0.38 ^b^	−0.19 ^b^	0.03 ^b^	1.00 ^b^

Note: *n*: number of subjects; SRH = self-reported health; SRA = self-reported anxiety; RT = reaction time; SD: standard deviation; AFE = area of the fitting ellipse; ^a^ = Spearman’s rank correlation coefficient; ^b^ = Pearson’s correlation coefficient.

**Table 5:** Results of the simple linear regression analysis for the effect of age on Poincaré parameters (*n* = 120).

Variable	R	R Square	Adjusted R Square	SE	F	*p*	β0	SE	*p*	95%LB	95%UB	β1	SE	*p*	95%LB	95%UB
**RT**	0.79	0.63	0.63	73.10	202.39	0.001	153.53	15	0.001	123.83	183.22	4.51	0.32	0.001	3.89	5.14
**SD_1_**	0.23	0.05	0.05	30.94	6.77	0.01	66.33	6.35	0.001	53.76	78.90	0.35	0.13	0.01	0.08	0.62
**SD_2_**	0.09	0.008	−0.0008	34.48	0.90	0.34	79.65	7.07	0.001	65.65	93.66	0.14	0.15	0.343	−0.15	0.44
**AFE**	0.13	0.02	0.01	17096.7	2.14	0.15	20003.73	3507.58	0.001	13057.77	26949.70	108.69	74.22	0.145	−38.28	255.66
**SD_1_/SD_2_**	0.32	0.10	0.10	0.20	13.72	0.001	0.82	0.04	0.001	0.74	0.91	0.003	0.001	0.001	0.001	0.01

Note: *n* = number of subjects; RT = reaction time; SD = standard deviation; AFE = area of the fitting ellipse; R = coefficient of correlation; R square = coefficient of determination; adjusted R square = effect size indicator; SE = standard error; F = value of F-test for overall significance; *p* = thresholds of statistical significance; β0 = the intercept parameter; β1 = the slope parameter; 95%LB = lower bound of the 95% confidence interval; 95%UB = upper bound of the 95% confidence interval.

**Table 6:** Results of the ordinal regression analysis for the effect of Poincaré parameters on SRH (*n* = 120).

Variable	Model Fit		Pseudo R-Square Nagelkerke	Parameter Estimates					
	Chi Square	*p*		Estimate	SE	Wald	*p*	95%LB	95%UB
**RT**	9.90	0.002	0.09	0.005	0.001	10.62	0.001	0.002	0.008
**SD_1_**	25.65	0.001	0.21	0.029	0.006	23.49	0.001	0.017	0.041
**SD_2_**	23.93	0.001	0.20	0.025	0.005	21.17	0.001	0.014	0.036
**AFE**	30.69	0.001	0.25	0.001	0.001	26.61	0.001	0.001	0.001
**SD_1_/SD_2_**	0.057	0.812	0.001	0.193	0.784	0.061	0.806	−1.343	1.729

Note: *n* = number of subjects; SRH = self-reported health; RT = reaction time; SD = standard deviation; AFE = area of the fitting ellipse; SE: = standard error; *p* = thresholds of statistical significance; 95%LB = lower bound of the 95% confidence interval; 95%UB = upper bound of the 95% confidence interval.

**Table 7:** Results of the multiple regression analysis for the effect of age and SRH on Poincaré parameters (*n* = 120).

Variable	R	R Square	Adjusted R Square	SE	F	*p*	Regression Equation
**RT**	0.80	0.63	0.63	73.32	100.74	0.001	z = 4.46 * x + 4.26 * y + 145.47
**SD_1_**	0.46	0.21	0.20	28.36	15.73	0.001	z = 0.16 * x + 14.70 * y + 39.55
**SD_2_**	0.44	0.19	0.18	31.29	13.69	0.001	z = −0.08 * x + 17.19 * y + 48.35
**AFE**	0.48	0.23	0.22	15182.26	17.68	0.001	z = −11.26 * x + 9296.89 * y + 3078.20

Note: *n* = number of subjects; SRH = self-reported health; RT = reaction time; SD = standard deviation; AFE = area of the fitting ellipse; R = coefficient of correlation; R square = coefficient of determination; adjusted R square = effect size indicator; SE: = standard error; F = value of F-test for overall significance; *p* = thresholds of statistical significance; x = the predictor variable (age); y = the predictor variable (SRH); z = the outcome variable.

## Data Availability

All data relevant to the study are included in the article.

## References

[1] Eckner J.T., Richardson J.K., Kim H., Lipps D.B., Ashton-Miller J.A. (2012). A novel clinical test of recognition reaction time in healthy adults. Psychol. Assess..

[2] Mercer V.S., Hankins C.C., Spinks A.J., Tedder D.D. (2009). Reliability and validity of a clinical test of reaction time in older adults. J. Geriatr. Phys. Ther..

[3] Baayen R.H., Milin P. (2010). Analyzing reaction times. Int. J. Psychol. Res..

[4] Reddy S., Eckner J.T., Kutcher J.S. (2014). Effect of acute exercise on clinically measured reaction time in collegiate athletes. Med. Sci. Sports Exerc..

[5] Nuri L., Shadmehr A., Ghotbi N., Moghadam B.A. (2013). Reaction time and anticipatory skill of athletes in open and closed skill-dominated sport. Eur. J. Sport Sci..

[6] Badau D., Baydil B., Badau A. (2018). Differences among three measures of reaction time based on hand laterality in individual sports. Sports.

[7] Tamm L., Narad M.E., Antonini T.N., O’Brien K.M., Hawk L.W., Epstein J.N. (2012). Reaction time variability in ADHD: A review. Neurotherapeutics.

[8] Iconaru E.I., Ciucurel C. (2020). Hand grip strength variability during serial testing as an entropic biomarker of aging: A Poincaré plot analysis. BMC Geriatr..

[9] Hultsch D.F., Macdonald S.W.S., Dixon R.A. (2002). Variability in reaction time performance of younger and older adults. J. Gerontol. Ser. B.

[10] Benton A.L. (1977). Interactive effects of age and brain disease on reaction time. Arch. Neurol..

[11] Aylward J., Valton V., Goer F., Mkrtchian A., Lally N., Peters S., Limbachya T., Robinson O.J. (2017). The impact of induced anxiety on affective response inhibition. R. Soc. Open Sci..

[12] Antonini T.N., Narad M.E., Langberg J.M., Epstein J.N. (2013). Behavioral correlates of reaction time variability in children with and without ADHD. Neuropsychology.

[13] Fishman M., Jacono F.J., Park S., Jamasebi R., Thungtong A., Loparo K.A., Dick T.E. (2012). A method for analyzing temporal patterns of variability of a time series from Poincaré plots. J. Appl. Physiol..

[14] Golińska A.K. (2013). Poincaré plots in analysis of selected biomedical signals. Stud. Logic Gramm. Rhetor..

[15] Guzik P., Piskorski J., Krauze T., Schneider R., Wesseling K.H., Wykretowicz A., Wysocki H. (2007). Correlations between the Poincaré plot and conventional heart rate variability parameters assessed during paced breathing. J. Physiol. Sci..

[16] Crenier L. (2014). Poincaré plot quantification for assessing glucose variability from continuous glucose monitoring systems and a new risk marker for hypoglycemia: Application to type 1 diabetes patients switching to continuous subcutaneous insulin infusion. Diabetes Technol. Ther..

[17] Satti R., Abid N.-U.-H., Bottaro M., De Rui M., Garrido M., Raoufy M.R., Montagnese S., Mani A.R., Rauofy M.R. (2019). The application of the extended Poincaré plot in the analysis of physiological variabilities. Front. Physiol..

[18] Sieciński S., Kostka P.S., Tkacz E.J. (2020). Heart rate variability analysis on electrocardiograms, Seismocardiograms and Gyrocardiograms on healthy volunteers. Sensors.

[19] Huo C., Huang X., Zhuang J., Hou F., Ni H., Ning X. (2013). Quadrantal multi-scale distribution entropy analysis of heartbeat interval series based on a modified Poincaré plot. Phys. A Stat. Mech. Appl..

[20] Roy S., Goswami D.P., Sengupta A. (2020). Geometry of the Poincaré plot can segregate the two arms of autonomic nervous system—A hypothesis. Med. Hypotheses.

[21] Günendi Z., Taskiran O.O., Beyazova M. (2005). What is the optimal repetition number in electromyographic reaction time studies?. Clin. Biomech..

[22] Stoet G. (2010). PsyToolkit: A software package for programming psychological experiments using Linux. Behav. Res. Methods.

[23] Stoet G. (2017). PsyToolkit: A novel web-based method for running online questionnaires and reaction-time experiments. Teach. Psychol..

[24] Fonseca N.T., Quesada J., Nolasco A., Melchor I., Moncho J., Pereyra-Zamora P., Lopez R., Calabuig J., Barber X. (2013). Self-rated health and mortality: A follow-up study of a Spanish population. Public Health.

[25] Jylhä M. (2009). What is self-rated health and why does it predict mortality? Towards a unified conceptual model. Soc. Sci. Med..

[26] Kimball S.M., Mirhosseini N., Rucklidge J. (2018). Database analysis of depression and anxiety in a community sample—Response to a micronutrient intervention. Nutrients.

[27] Woods D.L., Wyma J.M., Yund E.W., Herron T.J., Reed B. (2015). Factors influencing the latency of simple reaction time. Front. Hum. Neurosci..

[28] IBM Corp. (2011). IBM SPSS Statistics for Windows, Version 20.0, Released 2011.

[29] Contreras-Reyes J.E., Idrovo-Aguirre B.J. (2020). Backcasting and forecasting time series using detrended cross-correlation analysis. Physical A.

[30] Karmakar C.K., Khandoker A.H., Gubbi J., Palaniswami M. (2009). Complex correlation measure: A novel descriptor for Poincaré plot. Biomed. Eng. Online.

[31] Li X., Wong W., Lamoureux E.L., Wong T.Y. (2012). Are linear regression techniques appropriate for analysis when the dependent (outcome) variable is not normally distributed?. Investig. Opthalmol. Vis. Sci..

[32] Cohen J. (1988). Statistical Power Analysis for the Behavioral Sciences.

[33] Deary I.J., Der G. (2005). Reaction time, age, and cognitive ability: Longitudinal findings from age 16 to 63 years in representative population samples. Aging Neuropsychol. Cogn..

[34] Anstey K.J., Dear K., Christensen H., Jorm A.F. (2005). Biomarkers, health, lifestyle, and demographic variables as correlates of Reaction time performance in early, middle, and late adulthood. Q. J. Exp. Psychol. Sect. A.

[35] Blomkvist A.W., Eika F., Rahbek M.T., Eikhof K.D., Hansen M.D., Søndergaard M., Ryg J., Andersen S., Jørgensen M.G. (2017). Reference data on reaction time and aging using the Nintendo Wii Balance Board: A cross-sectional study of 354 subjects from 20 to 99 years of age. PLoS ONE.

[36] Fozard J.L., Vercruyssen M., Reynolds S.L., Hancock P.A., Quilter R.E. (1994). Age differences and changes in reaction time: The baltimore longitudinal study of aging. J. Gerontol..

[37] Porciatti V., Fiorentini A., Morrone M., Burr D.C. (1999). The effects of ageing on reaction times to motion onset. Vis. Res..

[38] Der G., Deary I.J. (2006). Age and sex differences in reaction time in adulthood: Results from the United Kingdom health and lifestyle survey. Psychol. Aging.

[39] Jain A., Bansal R., Kumar A., Singh K.D. (2015). A comparative study of visual and auditory reaction times on the basis of gender and physical activity levels of medical first year students. Int. J. Appl. Basic Med. Res..

[40] Dykiert D., Der G., Starr J.M., Deary I.J. (2012). Sex differences in reaction time mean and intraindividual variability across the life span. Dev. Psychol..

[41] Landauer A.A., Armstrong S., Digwood J. (1980). Sex difference in choice reaction time. Br. J. Psychol..

[42] Bienertova-Vasku J., Zlamal F., Nečesánek I., Konečný D., Vasků A. (2016). Calculating stress: From entropy to a thermodynamic concept of health and disease. PLoS ONE.

[43] Kallen V., Tahir M., Bedard A., Bongers B., van Riel N., van Meeteren N. (2021). Aging and allostasis: Using bayesian network analytics to explore and evaluate allostatic markers in the context of aging. Diagnostics.

[44] Tarabichi M., Antoniou A., Saiselet M., Pita J.M., Andry G., Dumont J.E., Detours V., Maenhaut C. (2013). Systems biology of cancer: Entropy, disorder, and selection-driven evolution to independence, invasion and “swarm intelligence”. Cancer Metastasis Rev..

[45] Kochan N.A., Bunce D., Pont S., Crawford J.D., Brodaty H., Sachdev P.S. (2017). Is intraindividual reaction time variability an independent cognitive predictor of mortality in old age? Findings from the Sydney Memory and Ageing Study. PLoS ONE.

[46] Moradi A., Esmaeilzadeh S. (2017). Simple reaction time and obesity in children: Whether there is a relationship?. Environ. Health Prev. Med..

[47] Smith A.P., Brice C., Leach A., Tiley M., Williamson S. (2004). Effects of upper respiratory tract illnesses in a working population. Ergonomics.

[48] Song Y.H., Ha S.-M., Yook J.S., Ha M.-S. (2019). Interactive improvements of visual and auditory function for enhancing performance in youth soccer players. Int. J. Environ. Res. Public Health.

[49] Easey K.E., Catling J.C., Kent C., Crouch C., Jackson S., Munafò M.R., Attwood A.S. (2018). State anxiety and information processing: A 7.5% carbon dioxide challenge study. Psychon. Bull. Rev..

[50] Diaper A., Nutt D.J., Munafò M.R., White J.L., Farmer E.W., E Bailey J. (2011). The effects of 7.5% carbon dioxide inhalation on task performance in healthy volunteers. J. Psychopharmacol..

[51] Gale C.R., Harris A., Deary I.J. (2016). Reaction time and onset of psychological distress: The UK Health and Lifestyle Survey. J. Epidemiol. Community Health.

